# Preparation and characteristics of carboxymethyl cellulose-based films embedding cinnamon essential oil and their application on mutton preservation

**DOI:** 10.3389/fnut.2025.1559833

**Published:** 2025-04-30

**Authors:** Han Wang, Peng Han, Yanghong Zhao, Lijuan Lu, Wenhui Qi, Kaixuan Zhao, Ying Shu, Zhisheng Zhang

**Affiliations:** ^1^College of Food Science and Technology, Hebei Agricultural University, Baoding, Hebei, China; ^2^Baoding Municipal People's Government, Baoding, Hebei, China; ^3^Shijiazhuang Food and Drug Inspection Center, Shijiazhuang, Hebei, China; ^4^Luopu Selenium Pigeon Industrial, Ltd., Luopu, China

**Keywords:** edible films, cinnamon essential oil, antibacterial activity, shelf life, mutton preservation

## Abstract

Here, the cinnamon essential oil (CEO) was distributed evenly in the carboxymethyl cellulose (CMC) matrix, and an edible film was fabricated to improve its physicochemical properties and extend the shelf life of mutton. The results revealed that the film had high visible light transmittance, ultraviolet-blocking properties, and satisfactory mechanical. Incorporating CEO enhanced the antibacterial activity against Escherichia coli and Staphylococcus aureus. When the CEO concentration was 1.5 g/L, the oxygen permeability of the film was the lowest, and mechanical properties were the highest. When the CEO increased, the thickness of the films increased, and the moisture absorption and water solubility of the film decreased. Compared with mutton covered without film or with control film, the film containing CEO more significantly reduced pH value, total volatile basic nitrogen, thiobarbituric acid reactive substances, and total bacterial count of mutton samples, improved the color of the meat, and extended the shelf life of the mutton during the 12-day storage at 4°C. These results demonstrate the potential of the developed CMC-based film in preserving meat.

## 1 Introduction

Human consumption of mutton has a long history. Mutton has the characteristics of high protein and low fat and is the main meat in various countries (1). However, mutton is extremely perishable (2). Thus, how to effectively delay spoilage and ensure the quality and safety of meat has become an important research topic in various countries (3, 4). Although freezing mutton at <-18° can effectively extend the shelf life of meat, ice crystals formed between muscle cells during freezing and recrystallization during storage can seriously affect the quality of meat (5, 6). At present, the public is also more inclined to buy fresh mutton. To preserve mutton quality while extending its shelf life, consumers predominantly rely on traditional polyethylene (PE) cling film for storage (7, 8). However, over 300 million metric tons of plastic waste are generated globally each year, and conventional PE packaging exhibits extremely low biodegradability, leading to long-term environmental persistence, while its microplastic byproducts pose significant risks to ecosystems and human health (9, 10). The global food packaging industry urgently requires sustainable transformation, making the development of biodegradable and multifunctional packaging materials a critical priority for the food sector's sustainable development.

Carboxymethyl cellulose (CMC), a chemically modified derivative of cellulose that ranks as Earth's most abundant renewable organic compound, is recognized as a promising alternative to conventional plastic packaging owing to its superior biodegradability and exceptional film-forming properties (11–13). Biodegradable packaging film prepared with CMC as the substrate has safety and environmental protection characteristics, demonstrates application value in reducing petroleum-based plastic usage, and can also extend the shelf life of food (14). Although this kind of film has certain mechanical properties, it has poor antioxidant and bacteriostatic ability and cannot effectively ensure the freshness of meat products (15–17).

Cinnamon essential oil (CEO), a natural plant extract containing bioactive components such as cinnamaldehyde and eugenol, has been scientifically validated for its antimicrobial and antioxidant properties (18, 19).

The incorporation of CEO into carboxymethyl cellulose (CMC)-based composite films represents an innovative strategy for developing multifunctional bioactive packaging. The composite film achieves material efficiency optimization by integrating CEO's dual antimicrobial and antioxidant functionalities, enabling a single-layer CMC structure to replicate the combined performance of conventional multilayer plastic barrier systems and synthetic preservatives. This innovation not only streamlines material composition but also circumvents recycling incompatibility issues inherent in hybrid multilayer packaging materials (20). Secondly, natural ingredients replace synthetic additives in the packaging system. The active compounds in cinnamon essential oil (CEO) demonstrate inhibitory effects against meat spoilage bacteria such as *Pseudomonas* and *Enterobacteriaceae* (21), while the natural formulation serves as a functional alternative to traditional synthetic antioxidants (e.g., BHT) and antimicrobial agents (e.g., potassium sorbate) (22). Notably, CMC-based films demonstrate superior environmental friendliness, achieving full biodegradation with non-ecotoxic degradation products, fundamentally solving the environmental pollution problem of microplastic residues from packaging films, and conforming to the eco-sustainable characteristics of modern food packaging fields (10, 23). Although the use of CMC and CEO in film production increases material costs in the short term, their capacity to extend food shelf-life and avoid material recycling costs demonstrates the long-term competitiveness of sustainable packaging in terms of environmental protection and economic benefits. However, its low solubility in the water phase and high sensitivity to oxygen and temperature limit its application in food (24). A previous study reports that adding CEO to carbohydrate-based film can enhance its antioxidant and antimicrobial properties (25, 26).

To overcome the mentioned problems, we added CEO to CMC-based film, adsorbed CMC on the essential oil-water interface, distributed CEO in the form of tiny oil droplets uniformly in the film, and prepared an edible active film. The films were characterized, and the effects of the concentration of the CEO on the CMC film were analyzed. The edible biofilm was applied to the packaging of fresh mutton to explore the impact of the film on the quality change of mutton in the process of corruption and to demonstrate the feasibility of CEO-CMC film to extend the shelf life of mutton. This study provides a theoretical basis for applying bio-packaging film in meat.

## 2 Material and methods

### 2.1 Materials

The CEO (containing 80% cinnamaldehyde and 6.25% eugenol) was supplied by FLOWERS & HERBS Co. Ltd., Beijing, China, and produced by steam distillation of fresh cinnamon tree branches, leaves, and bark. *S. aureus* and *E. coli* were provided by Strain Preservation Laboratory of Hebei Agricultural University. Nutrient agars were produced in AoBoXing Bio-tech Co. Ltd., China. All the reagents used were purchased from Aladdin Reagent Co. Ltd., China. All of the chemical reagents were used without further purification and were analytical grade.

### 2.2 Preparation of films

CMC (1 g) was added to 100 mL deionized water and stirred at 80°C with a D130 dispersion machine (WIGGENS, Straubenhardt, Baden, Germany) 800 r/min for 20 min to obtain a film-forming liquid. After cooling to room temperature, CEO was added to the film-forming liquid and pre-emulsified with the dispersing machine at 10,000 r/min for 5 min. The pre-emulsified mixture was homogenized using a VCX750 ultrasonic processor (SONICS, Newtown, Connecticut, USA) in an ice-water bath at 300 W power in a pulse mode (on 5 s, and off for 5 s) for 1min. Pour the emulsion into an acrylic film-forming tank (120 mm × 120 mm) and let it stand in an oven (DHG-9143BS-III, CIMO, Shanghai) at 60°C for 24 h. The dried film was stripped and stored in a dryer at a temperature of 25°C and a relative humidity (RH) of 43%. The formulation of CMC-based films was list in Table 1.

**Table 1:** The formulation of CMC-based films.

**Films**	**CMC concentration (g/L)**	**CEO concentration (g/L)**
F0	10	0
F1	10	1.5
F2	10	3
F3	10	4.5
F4	10	6
F5	10	7.5

### 2.3 Determination of physicochemical indexes of the films

#### 2.3.1 Thicknesses

A 221 digital Micrometer (SanLiang Digimatic Micrometer, Shanghai) was used to measure the thickness of ten randomly selected locations of a film. The results were recorded, and the average value was taken as the thickness of the film.

#### 2.3.2 Water solubility and moisture absorption

The WS and MA were measured according to a previous study with some modifications (27). One piece of the film (2.0 cm × 2.0 cm) was placed at room temperature for 24 h, weighted (W_1_). The film was then dried in an oven (DHG-9143BS-III, CIMO, Shanghai) at 80°C and weighted again (W_2_). The MA of the film was calculated using the following equation.


(1) $$\begin{array}{l} MA\left(\%\right)=\frac{W_{1}-W_{2}}{W_{1}}\times 100 \end{array}$$

The film was left at room temperature for 24 h and weighed (W_3_). Then, it was put into 60 mL of deionized water and stirred at 300 rpm at 37°C until completely dissolved. The dissolution time t was recorded. The WS of the film was represented by the mass of the dissolved film per second and calculated using the following equation.


(2) $$\begin{array}{l} WS\left(g/s\right)=\frac{W_{3}}{t} \end{array}$$

#### 2.3.3 Light transmission

The film was cut into a small piece of 1.0 cm × 4.5 cm and then stuck to the inner side of a cuvette at room temperature. The light transmittance of the film at wavelengths from 200 nm to 800 nm were measured by an ultraviolet-visible spectrophotometer (UV-2600, Shimadzu, Kyoto, Japan).

#### 2.3.4 Color parameters

The color parameters of the films were measured following the method described in a previous study (28). The CR-400 color difference meter (Konica Minolta Sensing Inc., Tokyo, Japan) was calibrated with a white reflector standard plate. Brightness (L^*^), redness (a^*^), and yellowness (b^*^) of the film were measured using the calibrated color difference meter. Five points of the film were randomly measured and averaged.

#### 2.3.5 Mechanical properties

The mechanical properties of the films were measured following the method described in a previous study with some modifications (27). The tensile stress (TS) and elongation at break (EAB) of the films were measured by a TMS-Pilot texture analyzer (FTC, Leesburg, Virginia, USA) at room temperature. The film samples were cut into pieces of 15 mm × 80 mm. The spacing of tensile probes was 50 mm, and the strain rate was 200 mm/min. Each sample was measured three times and averaged.

#### 2.3.6 Oxygen permeability

The OP of the films was measured following the method described in a previous study with some modifications (29). A VAC-VBS gas transmission rate tester (Labthink, Jinan, Shandong) was used to measure the OP of the films. The replacement time of experimental gas was 60 s, and the gas pressure was 1.01 kgf/cm^2^. Each sample was measured three times and averaged.

#### 2.3.7 Scanning electron microscopy

The microstructure of the films was measured following the method described in a previous study (30). The films were immersed in liquid nitrogen and fractured. After gold coating, they were observed via SEM (Prisma E, Thermo Scientific, Waltham, MA, USA) at an accelerating voltage of 30 kV.

#### 2.3.8 Fourier transform infrared spectrometer

A NI10 FT-IR (Thermo Scientific, Waltham, MA, USA) was used to measure the chemical structures of the films. FT-IR spectra were recorded using attenuated total reflection mode with a wavenumber ranging from 500 cm^−1^ to 4,000 cm^−1^.

#### 2.3.9 X-ray diffraction

The crystal structure and the crystallinity of the films were measured as described in previous studies (31, 32). The films were measured using a D2PHASER X-ray diffractometer (Bruker, Karlsruhe, Baden, Germany) equipped with the Cu-Ka radiation with a grazing incident angle from 0° to 50°. The test current was set at 50 mA, and the voltage was 40 kV. The crystallinity of the films was calculated with Origin 2018 (OriginLab, Northampton, MA, USA).

### 2.4 Evaluation of antibacterial activity

The antibacterial activity of the films was evaluated following the method described in a previous study with some modifications (33). The films were evaluated using the agar disk diffusion method in two bacteria, namely *E. coli* and *S. aureus*. Microorganisms were inoculated in nutrient broth 37°C for 24 h and diluted to 0.5 McFarland standards. The 0.5 mL microbial suspension was evenly coated on the AGAR medium plate, the film (1.0 cm in diameter) was sterilized by ultraviolet for 2 h and placed at the midpoint of the AGAR medium, and then cultured at 37°C for 12 h to observe whether a bacteriological zone was formed under or around the film.

### 2.5 Soil burial degradation test

The Soil Burial Degradation Test was conducted following the methodology outlined in prior research (34). 30 mm × 30 mm films were embedded in soil-containing culture plates. Daily mist irrigation cycles were implemented, with degradation monitoring performed at three-day intervals.

### 2.6 Mutton wrapping

Small-tailed Han Sheep 10–15 months old were slaughtered, and the hind leg meat was taken for experiment within 2 h. After the mutton was divided (the meat sample size was 2 cm × 2 cm × 1 cm if not specified), it was wrapped in a film and placed in a sterile petri dish. The samples were refrigerated at 4°C (I-36VL, Percival Scientific, Perry, Iowa, USA) and were analyzed every 2 days for physical, chemical, and microbiological analysis.

#### 2.6.1 pH

A 205 pH meter (testo, Lenzkirch, Baden, Germany) was inserted into the center of the mutton sample to measure the pH value. The experiment was repeated three times to take an average value.

#### 2.6.2 Color parameters

The color parameters of the mutton samples were measured following the method described in Section 2.3.4.

#### 2.6.3 Total volatile basic nitrogen

The TVB-N of the mutton samples was measured following the method described in a previous study (1). The TVB-N content of mutton was measured at the automatic Kjeldahl nitrogen determination apparatus (K1100, Hanon, Jinan, Shandong).

#### 2.6.4 Thiobarbituric acid reactive substances

The TBARS of the mutton samples was evaluated following the method described in a previous study (1). The milligrams of malonaldehyde (MDA) was converted to TBARS number per kilogram of mutton (mg MDA/kg mutton).

#### 2.6.5 Total viable counts

The TVC of the mutton samples was evaluated following the method described in a previous study (2). After the mutton was chopped with a sterilized scalpel, 1 g of ground mutton was homogenized in 10 mL normal saline for 1 min. The resulting mixture was diluted with normal saline at a 10-fold gradient. After selecting the appropriate gradient, 1 mL diluent was coated on a plate counting AGAR and cultured at 37°C for 24 h. The microbial count unit was log_10_ CFU/g.

#### 2.6.6 Electronic nose analysis

The Electronic Nose analysis was conducted following the methodology outlined in prior research (35). The samples were placed into 50 mL sealed vials and equilibrated at room temperature for 2 h. Analysis was performed using an electronic nose (CNose-18, Bosin Tech, Shanghai) with a detection time of 60 seconds and a gas flow rate of 120 mL/min. Signal values from 50 to 60 seconds were selected for data processing.

### 2.7 Statistical analysis

All experiments were repeated three times, and the data were presented as mean ± standard deviation (SD, *n* = 3). Experimental data were statistically analyzed through single-factor variance partitioning (ANOVA). Analysis of variance and significant difference (*p* ≤ 0.05) between treatments were determined using Duncan's multiple range test by the SPSS 21.0 (IBM, Armonk, NY, USA). Following significance testing (*p* ≤ 0.05), Pearson correlations (|r| ≥ 0.5) quantified linear associations between composite film parameters and mutton storage indices (36).

## 3 Results and discussion

### 3.1 Light properties of the films

**Figure 1:**
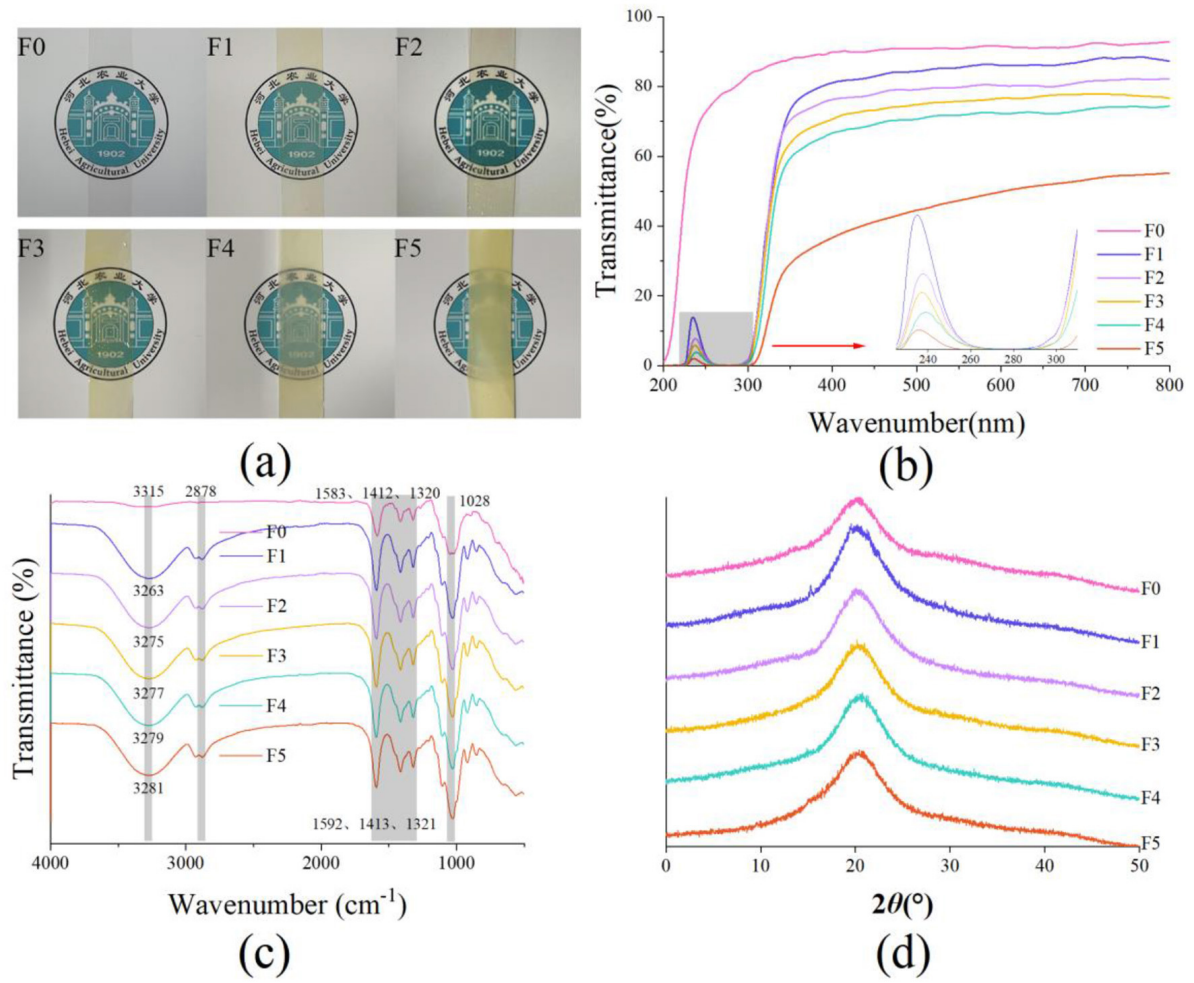
The photographs **(a)**, ultraviolet and visible light transmittance **(b)**, FT-IR spectra **(c)**, and XRD spectra **(d)** of the films without cinnamon essential oil or added with cinnamon essential oil (CEO). CEO concentrations were 0, 1.5 g/L, 3.0 g/L, 4.5 g/L, 6.0 g/L, and 7.5 g/L for F0, F1, F2, F3, F4, and F5, respectively.

Figure 1a shows the appearance of the films. F0 without CEO was colorless and transparent. With the increase in CEO from 1.5 to 7.5 g/L, the film gradually turned yellow, and its transparency was reduced. With the increase in CEO concentration, the blending performance of CEO and CMC deteriorates, resulting in a rough surface of the film, easier refraction or scattering of light through the film, and reduced light transmission. A similar result was reported that the visual color of films was gradually changed to yellow with increase in CEO (37).

The light transmittance of a film is an important property that determines whether the film can protect the sample from light, especially ultraviolet radiation. The ultraviolet and visible transmittance spectra (200–800 nm) of the films is shown in Figure 1b. F0 had high light transmittance in both ultraviolet and visible wavelengths. When the wavelength increased from 200 nm to 300 nm, the transmittance of F0 rapidly increased from 0 to about 83.10%, and then, with the increase in wavelength, the transmittance of the film remained constant (above 83.10%). A similar transmittance value of pure CMC films was reported in a previous study (38). The transmittance of F0 at 230 nm wavelength was 58.63%. The films containing CEO showed a strong transmittance peak in ultraviolet-light range at 230 nm. At this wavelength, the addition of CEO-1.5 g/L, CEO-3.0 g/L, CEO-4.5 g/L, CEO-6.0 g/L, and CEO-7.5 g/L exhibited a significant reduction in the maximum transmittance value of the films decreasing from to 8.08%, 2.78% 2.15%, 1.02%, and 0.80%. The light transmittance value of films also showed an ultraviolet absorption peak at around 280 nm. In the ultraviolet-light range (T280), the light transmittance value of F0 was 79.11% and decreased from 0.10% to 0.01% after the incorporation of OEO (from I.5 g/L to 7.5 g/L). The above result indicated that the ultraviolet barrier properties of the film significantly improved with the addition of CEO, and a significant improvement was observed with an increase in CEO concentration. This is because the phenolic compounds in CEO have absorption peaks in the ultraviolet region (25). The higher the concentration of CEO, the higher the content of phenolic compounds, the more obvious the absorption peak.

The films did not show any absorption peak in the visible light range. The transmittance value of F0 was 91.50% in visible light at 660 nm (T660). The transmittance values were significantly reduced with the CEO concentration increased from 1.5 to 7.5 g/L. The T660 of the film decreased to 86.25%, 79.85%, 77.49%, 72.67%, and 52.41% after adding CEO 1.5 g/L, 3.0 g/L, 4.5 g/L, 6.0 g/L, and 7.5 g/L, respectively. A similar result was reported that the transmittance value in the visible light range of a CMC-based film was decreased to about 53% after adding 3% of CEO (39). The transparency reduced with the increase in CEO concentration might be due to the obstruction of the light path by CEO droplets making it easier for lights to refract or scatter through the film, as confirmed by visual appearance and SEM results.

The color parameters of the films without CEO or with different CEO concentrations are shown in Table 2. The L^*^, a^*^, and b^*^ values were 93.78, −0.08, and 3.29, respectively. The F0 was colorless and transparent, but the films containing CEO displayed yellow and became darker with the increase of CEO concentrations and the decrease of the L^*^ value. After adding 7.5 g/L CEO, the a^*^ values of the film decreased from −0.08 to −3.70. The a^*^ values of the films were all negative, and their absolute values gradually increased, indicating enhanced green chromaticity with an increase in CEO concentration. The b^*^ values were all positive, indicating that the films were yellow. The lowest b^*^ value (3.29, 3.87, and 5.43) was displayed by F0, F1, and F2, followed by F3 and F4, while the maximum value (31.70) was shown by F5. The results of a previous study on the effect of CEO on the color of sodium caseinate film were consistent with our results (40). The CEO is dark yellow, so the film turns yellow with the increase in CEO concentration.

**Table 2:** Thickness, OP, WS, MA, mechanical properties and color properties (L^*^, a^*^, and b^*^) of films without CEO or with different CEO concentration.

**Films**	**Thickness (μm)**	**OP (cm^3^mm^−2^d^−1^ Pa^−1^ × 10^−11^)**	**WS (g/s × 10^5^)**	**MA (%)**	**TS (MPa)**	**EAB (%)**	L^*^	a^*^	b^*^
F0	16.10 ± 0.94^a^	3.99 ± 0.03^b^	64.14 ± 1.42^d^	9.89 ± 0.23^d^	40.53 ± 2.27^c^	12.31 ± 0.65^d^	93.78 ± 0.06^c^	−0.08 ± 0.01^a^	3.29 ± 0.01^a^
F1	16.81 ± 0.70^a^	2.16 ± 0.07^a^	64.05 ± 4.45^d^	5.22 ± 0.32^c^	81.34 ± 0.41^d^	13.66 ± 0.76^e^	93.83 ± 0.11^c^	−1.13 ± 0.01^a^	3.87 ± 0.11^a^
F2	18.30 ± 0.94^b^	4.05 ± 0.11^b^	43.83 ± 1.83^c^	4.38 ± 0.21^b^	79.67 ± 0.53^d^	10.73 ± 0.31^c^	92.77 ± 0.54^b^	−0.39 ± 0.08^a^	5.43 ± 0.50^a^
F3	19.25 ± 1.23^bc^	4.72 ± 0.12^c^	36.68 ± 3.05^b^	3.69 ± 0.35^a^	40.87 ± 1.24^c^	9.65 ± 0.16^b^	92.37 ± 0.39^b^	−1.96 ± 0.30^b^	13.61 ± 0.95^b^
F4	20.03 ± 1.17^c^	5.18 ± 0.29^d^	26.36 ± 1.57^a^	3.54 ± 0.14^a^	25.59 ± 0.11^b^	7.01 ± 0.04^a^	89.51 ± 0.83^a^	−2.37 ± 0.30^c^	26.01 ± 2.73^c^
F5	22.60 ± 1.67^d^	5.66 ± 0.14^e^	24.66 ± 0.44^a^	3.35 ± 0.21^a^	20.96 ± 3.00^a^	6.24 ± 0.23^a^	89.91 ± 0.15^a^	−3.70 ± 0.17^d^	31.70 ± 1.43^d^

Data were presented as mean ± standard error. CEO concentrations were 0, 1.5 g/L, 3.0 g/L, 4.5 g/L, 6.0 g/L, and 7.5 g/L for F0, F1, F2, F3, F4, and F5, respectively. ^a−*e*^Different superscripts indicate significant differences in the same column (*P* ≤ 0.05).

### 3.2 Thickness and OP properties of the films

The thickness of the films is shown in Table 2. When a small amount of CEO (1.5 g/L) was added, the thickness of the film was not significantly different from that of F0. With the increase of CEO concentration from 1.5 to 7.5 g/L, the thickness of the films gradually increased from 16.81 μm to 22.60 μm. Previous results on the effect of CEO on properties of chitosan-carboxymethyl cellulose films are consistent with ours (41). As a bio-based plasticizer of the film, a small amount of CEO can increase the molecular interaction force between molecules of the films and make the molecular structure denser. Further increasing the amount of CEO will increase the density and size of the pore, and the thickness of the film will increase. This is similar to the conclusions of other studies on CMC thin films (27).

The thickness and OP of the films are shown in Table 2. The OP value of F0 was 3.99 cm^3^mm^−2^d^−1^ Pa^−1^ × 10^−11^, which decreased with the addition of 1.5 g/L CEO (F1, 2.16 cm^3^mm^−2^d^−1^ Pa^−1^ × 10^−11^) and then increased gradually to 5.66 cm^3^mm^−2^d^−1^ Pa^−1^ × 10^−11^ (F5). A small amount CEO makes the molecular structure of the film denser and the gas shuttle path more curved and complicated, which increases the non-polar oxygen molecules barrier of the film (38). This result also may be due to the high antioxidant capacity and oxygen trapping ability of CEO, which also increases the difficulty of gas passage and reduces the OP value (25, 39). When the thickness is more than 18.30 μm, the film structure can be destroyed, the shuttle path of the gas is expanded, and the OP value increases significantly. A similar conclusion is reached for the effect of CEO on sodium starch octenylsuccinate-based Pickering emulsion film (15).

### 3.3 WS and MA of the films

The WS and MA of the films are shown in Table 2. With the CEO concentration increased from 0 to 7.5 g/L, the WS of the films decreased from 64.14 to 24.66 (g/s × 10^5^), and the MA of the films decreased from 9.89% to 3.35%. Pure CMC films can be completely dissolved in water. CEO is strong hydrophobicity. After it is added to the CMC film, the CEO obstructs the diffusion of water molecules in CMC films while destroying the network structure of CMC films. The structure of the films then became loose, and the density decreased, causing a downward trend. It may also be due to the interaction of hydroxyl groups in each film component after the CEO added the film, resulting in the reduction of free hydroxyl groups interacting with water molecules of the film (27). The composite film composed of CMC and other essential oils also has a similar phenomenon (15, 28).

### 3.4 Mechanical properties of the films

The mechanical properties of the films are shown in Table 2. With the increase in CEO concentration, the TS and EAB of the film showed a trend of increasing and then decreasing. When the concentration of CEO in the films increased from 0 to 7.5 g/L, the TS of the film increased from 40.53 MPa (F0) to 81.34 MPa (F1) and then gradually decreased to 20.96 (F5). EAB increased from 12.31% (F0) to 13.66% (F1) and then decreased to 6.24% (F5). Studies have shown that bio-based plasticizers can enhance the mechanical properties of films (38, 42). A previous study also found that adding a small amount of CEO (5%-20%) to poly (vinyl alcohol) films can increase their mechanical strength (43). The CEO can form an electrostatic attraction with the CMC, increasing the liquidity of the CMC chains. Therefore, the addition of low-dose CEO can be used as a bio-based plasticizer to improve the mechanical properties of the films. However, increasing the amount of CEO will increase the pore size and density of the films, reduce the cohesion and continuity of the CMC network structure, reducing of the intermolecular force. Thus, the mechanical properties of the films reduced with the increase in CEO concentrations. Similar results are reported in previous studies (44).

### 3.5 Molecular interactions of the films (FT-IR)

The FT-IR spectra of the films are shown in Figure 1c. The stretching vibration peak of O-H was at 3,315 cm^−1^. The wide peak near 2,900 cm^−1^ represented the asymmetric stretching vibration peak formed by C-H stretching (45). The peaks at 1,583 cm^−1^ and 1,412 cm^−1^ were the asymmetric and symmetrical stretching vibration peaks of COO-. The peak at 1,320 cm^−1^ was the bending vibration peak -OH. The peak at 1,105 cm^−1^ was the vibration peak of glycosidic bonds in polysaccharides (45). The stretching vibration peak of C-O is at 1,028 cm^−1^.

Compared with F0, the peak strength of O-H stretching vibration increased and moved to a lower wavelength after adding CEO. This result is due to the presence of phenols and alcohols in CEO. After the addition of CEO concentration, O-H groups increased, O-H bonds between phenols, alcohols, and water molecules formed, and the intermolecular interactions were enhanced (46). Due to the stretching of C-H, two peaks appeared near 2,900 cm^−1^ (47). The peak strength of the C-H expansion of CEO film (F1-F5) increased. The increase in aldehyde and lipid CH_2_ groups in the films could be due to the addition of cinnamaldehyde, increasing the amount of C-H (43, 46). The peaks at 1,583 cm^−1^, 1,411 cm^−1^, and 1,320 cm^−1^ were the characteristic peaks of CMC. The stretching vibration of cinnamaldehyde C=O and aromatic C=C groups overlapped with the COO-. So, the intensity of the three peaks increased slightly (46). In addition, phenols and alcohols in the CEO could also increase the peak strength of the C-O stretching vibration of the films (48).

With the increase in CEO concentration, the O-H stretching vibration peak moved to a high wavelength, indicating that the hydrogen bond between O-H in the films decreased, and the intermolecular force decreased. Thus, the dense structure of the film was destroyed. No wavelength deviated between the asymmetric and symmetrical stretching vibration peaks of COO-, which indicated that the change of CEO concentration did not affect the chemical structure of CMC molecules.

### 3.6 XRD patterns of the films

The XRD spectra of the films are shown in Figure 1d. The F0 showed a broad diffraction peak at 20° and the crystallinity was about 17%, indicating a low crystalline structure of CMC (12, 17). After the addition of CEO, no new diffraction peaks were formed, and no new crystal regions appeared in the films with CEO, indicating that the addition of CEO did not add a new crystalline structure to the films. Compared with F0, the diffraction peak of F1 was sharper, the area was larger, and the crystallinity (about 26%) was increased. Other studies also have similar result (49). This result might be due to heating or ultrasound attacking the amorphous region of the CMC, or it might be due to the CEO acting as a nucleating agent, which improved the density of the film. With the increase in CEO concentration, the diffraction peaks of F2, F3, F4, and F5 had no significant difference but were lower than that of F1. The crystallinity of the films (F2, F3, F4, and F5) was about 24%, lower than that of F1. The high concentration of CEO decreased the crystallinity of the films slightly. Similar results have also been reported in other literature (32). This result might be because excessive CEO weakened the regular arrangement between the CMC molecules.

### 3.7 Morphology film

**Figure 2:**
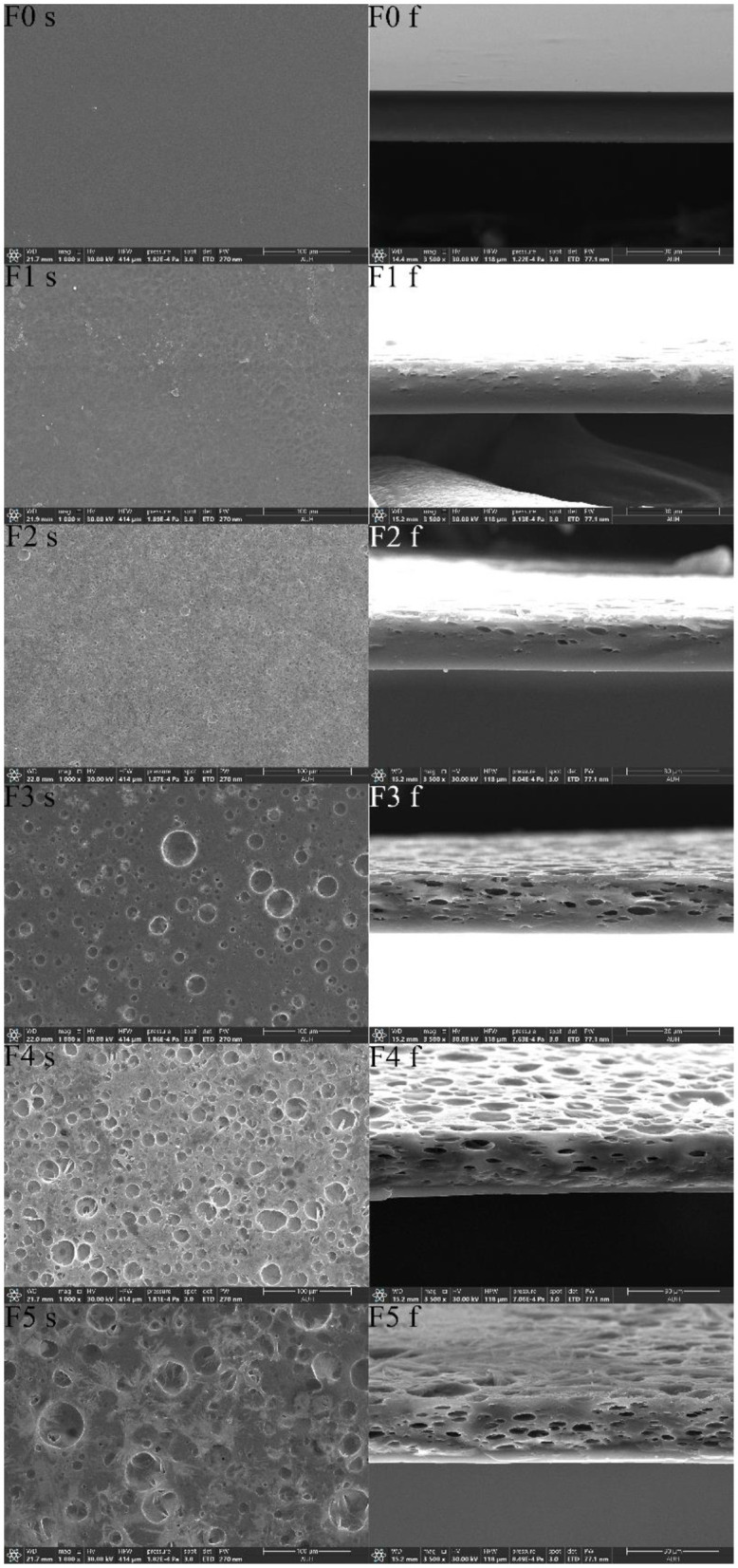
SEM images of surfaces and fractures of the films without CEO or with different CEO concentrations. CEO concentrations were 0, 1.5 g/L, 3.0 g/L, 4.5 g/L, 6.0 g/L, and 7.5 g/L for F0, F1, F2, F3, F4, and F5, respectively. s and f are the surface and fracture morphology of the film, respectively.

The surface and fracture morphology of the films could have an impact on the optical properties, mechanical properties, thickness, and OP of the films. The SEM images of the surface and fracture morphology of the films are shown in Figure 2. The surface of the film without CEO was smooth and flat, the cross-sections were relatively flat, and the structure was dense. With the increase in CEO concentration, the surface of the films became rough, holes and the number of pores in the cross-section became more and larger, and the structure became loose. During the air-drying process, emulsion droplets floated up, increasing porosity in the upper layer of F1 and F2 fracture morphology. When the concentration of CEO was 4.5 g/L, the emulsion droplets coalesced, resulting in huge pores on the surface of F3. When the concentration of CEO reached 6.0 g/L, demulsification occurred, and CEO precipitation occurred, resulting in irregular pore edges in F4 and F5. A previous study finds similar SEM images produced on the surface of the films containing CEO (49).

The increase in film thickness could be explained by the increased pore size in the films (Table 2). The continuous decline in the light transmittance of the films (Figure 1b) could be explained by the increase in the roughness of the film surface and the increase in the porosity. After demulsification, the CEO severely hindered the light path, which could explain a significant decrease in the light transmittance of F5. The decrease in film structure density of fracture morphology in F3, F4, and F5 could explain the decrease in mechanical properties and OP of the films.

### 3.8 Antibacterial activity of the films

**Figure 3:**
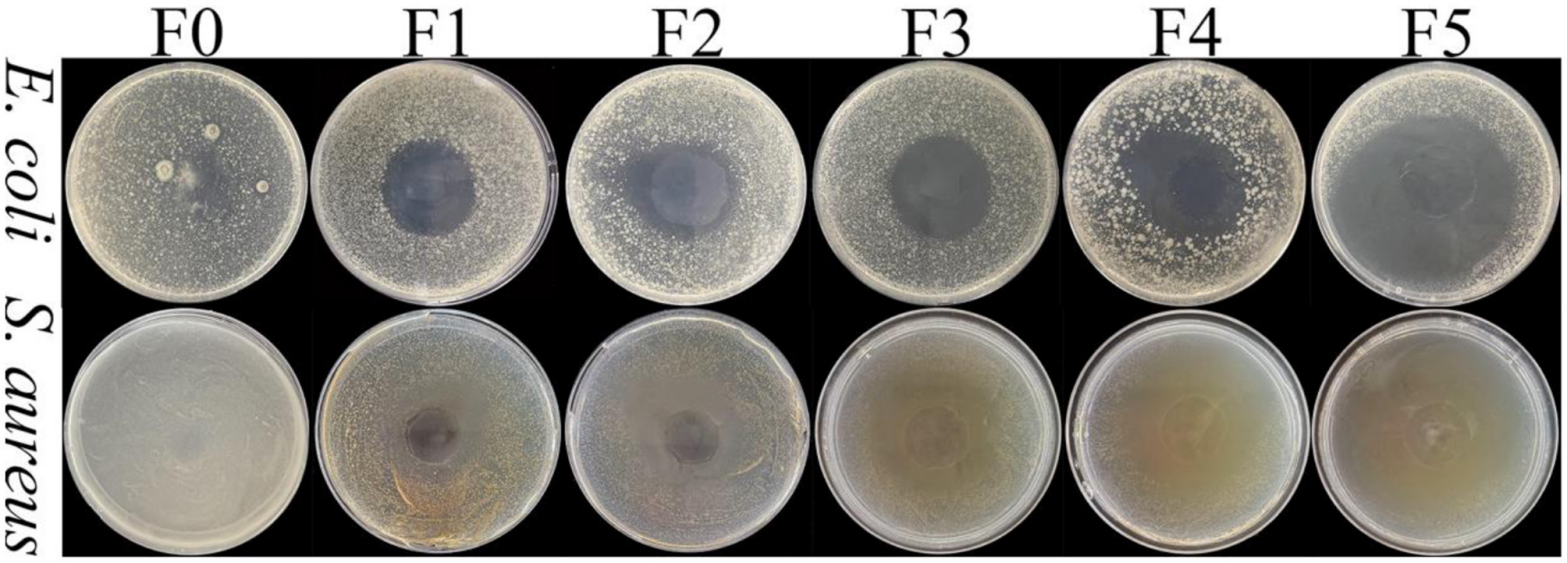
Antimicrobial assay of the films without CEO or with different CEO concentrations against *E. coli* and *S. aureus*.

*E. coli* is the common gram-negative bacteria, and *S. aureus* is the common gram-positive bacteria. The antibacterial abilities of the films are shown in Figure 3. F0 had no inhibitory effect on the two bacteria, and the bacteria would gather in the gel-like substance formed after the film absorbed water to form a large colony. After the addition of the CEO, the films had a noticeable antibacterial effect on *E. coli* and *S. aureus*, and antibacterial bands could be observed clearly. Similar results are reported in studies; with the increase of CEO concentration, the diameter of the antibacterial zone gradually expands (50). The main extracts of CEO are cinnamic aldehyde, eugenol, and linalool, and its antibacterial performance can be attributed to the high proportion of phenols and aldehydes. These lipophilic substances can penetrate the cell membrane, induce the cell membrane to self-aggregate into clumps, destroy the cell membrane structure, disrupt the energy metabolism and enzyme system, and thus produce cytotoxic effects on living microorganisms (51). In addition, cinnamaldehyde increases the expression of apoptosis-related genes.

After adding CEO, the films had better antibacterial activity against *S. aureus* than *E. coli*. The main component of the cell wall of gram-positive bacteria is peptidoglycan, and that of gram-negative bacteria is lipopolysaccharide. The hydrophobic substances of CEO easily pass through gram-positive cell walls, while lipopolysaccharides in the outer membrane of gram-negative cell walls make the cell surface hydrophilic and resistant to hydrophobic CEO. Similar results have also been reported in other literature (33). These results could also be supported by Supplementary Table S1.

### 3.9 Biodegradation

**Figure 4:**
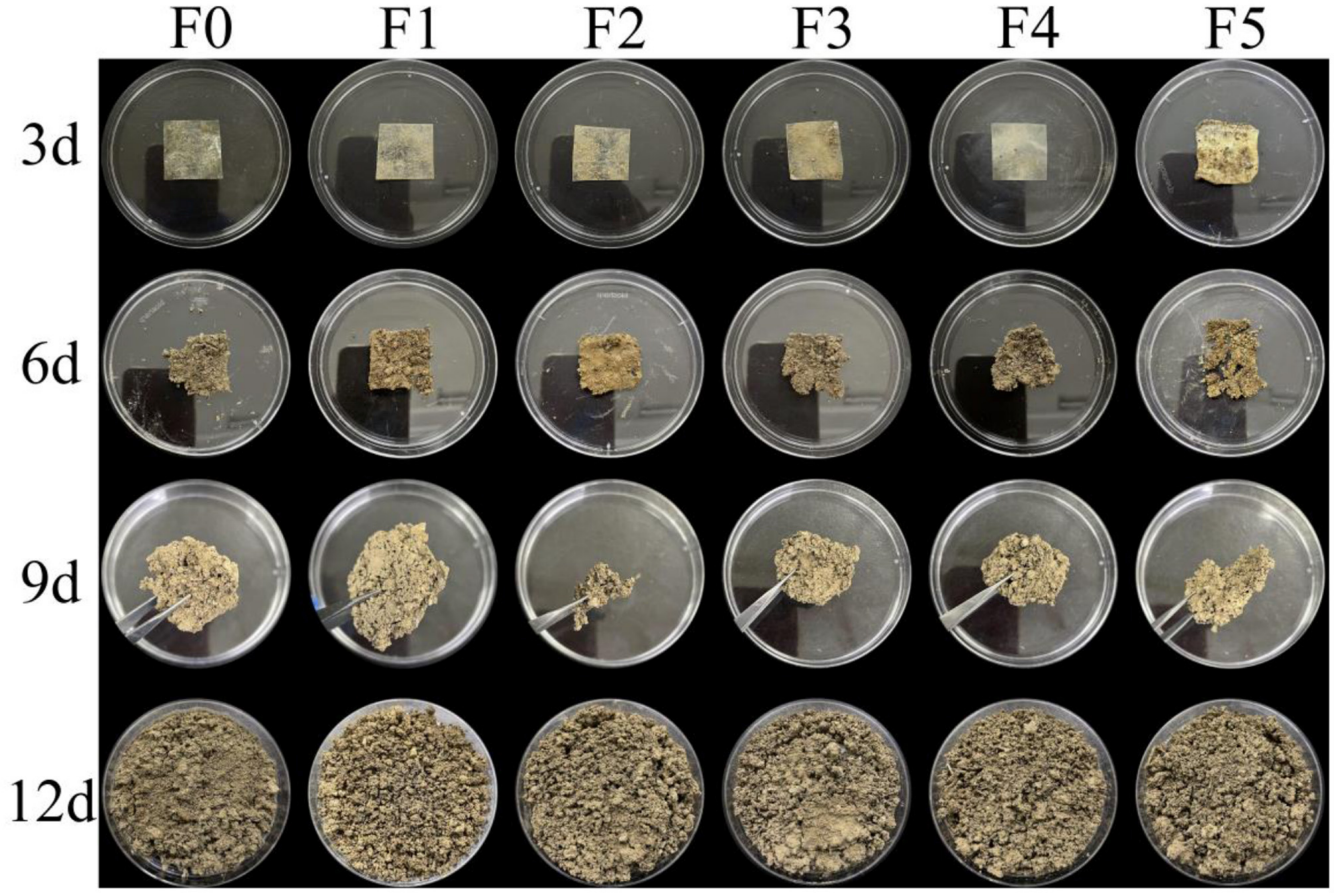
The soil burial degradation test of the films without CEO or with different CEO concentrations. CEO concentrations were 0, 1.5 g/L, 3.0 g/L, 4.5 g/L, 6.0 g/L, and 7.5 g/L for F0, F1, F2, F3, F4, and F5.

To evaluate the composite membrane's biodegradability, films F0–F5 were subjected to soil burial degradation testing over 0–12 days (Figure 4). All samples fully degraded within 12 days, confirming the film's exceptional biodegradability and sustainable biomass utilization. Degradation rates showed no correlation with CEO loading, which may be attributed to insufficient CEO concentrations or accelerated CMC matrix fragmentation via soil moisture-induced swelling/rupture, masking potential microbial activity modulation. Consistent findings were reported by Dong et al. (34).

### 3.10 Mutton wrapping

After the films were covered on the surface of the mutton, the evaluation of the meat quality parameters was conducted at 0, 2, 4, 6, 8, 10, and 12 days at 4°C. Sample groups were the mutton covered with F0, F1, F3, and F5, which were shorted for M0, M1, M3, and M5, respectively. The mutton covered without film was named as Control.

#### 3.10.1 Color parameters

**Figure 5:**
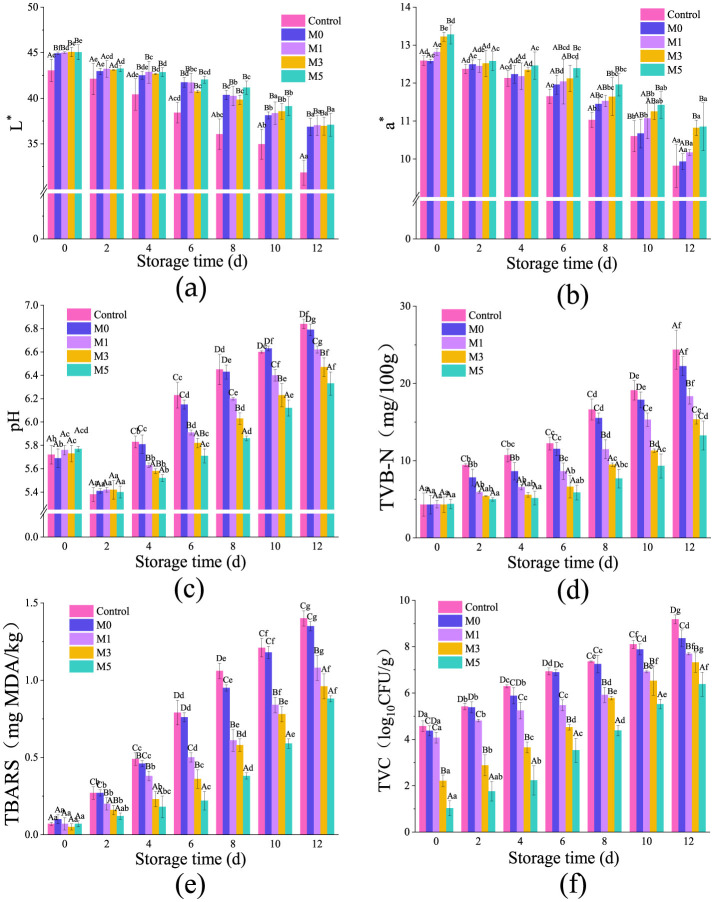
The color parameters [**(a)** for L* and **(b)** for a*], pH value **(c)**, TVB-N **(d)**, TBARS **(e)**, and TVC **(f)** values of mutton at time period of 12 days. The sample groups are the mutton covered with F0, F1, F3, and F5, which were shorted for M0, M1, M3, and M5, respectively. The mutton covered without film was shorted as Control. Different lowercase letters indicated that the average value of the same mutton treatment group is significantly different in different storage times (*p* ≤ 0.05). Different capital letters indicated that the average value of different mutton treatment groups on the same day is significantly different (*p* ≤ 0.05).

The color of mutton is mainly related to myoglobin in meat, which is roughly divided into oxygenated myoglobin (bright red), deoxygenated myoglobin (purplish red), and metmyoglobin (brown). Due to the decrease in oxygen content, the longer the mutton is stored, the higher the content of ferrimyoglobin (1). Thus, the value of L^*^ (brightness) and a^*^ (redness) of the mutton changed (Figures 5a, b).

As shown in Figure 5a, with the extension of time, the L^*^ value of mutton in the Control group decreased from 43.05 on day 0 to 31.84 on day 12. The decrease in the L^*^ value of M0 was delayed. After adding different concentrations of CEO, the L^*^ values of mutton in the M1, M3, and M5 groups were not significantly different from those in the M0 group. Guo claims that the decrease in L^*^ value was related to the content of metmyoglobin (52). Dal Bosco believed that the decline in L^*^ value was related to the increase in pH and the change of the isoelectric point of protein (53). However, some literature showed an opposite trend: the water retention of meat decreased, the internal bound water was transformed into free water and migrated to the surface, the light reflection ability was enhanced, and the L^*^ value increased (1). The opposite conclusion in this study may be that CMC film has strong water absorption and absorbs the water on the surface of the meat, so the value of L^*^ decreases.

The value of a^*^ represents the freshness of the meat quality, and the change of this value is mainly related to the change of oxygenated myoglobin. As can be seen from Figure 5b, a^*^ values of mutton all decreased with the extension of storage time. During the same storage period, with the increase of CEO concentration in the film, the a^*^ value of mutton showed an increasing trend. Similar results were found in other literature (54). At the 0 day, the a^*^ values of mutton in the M3 and M5 groups were significantly higher than those in the Control, M0, and M1 groups. This result may be related to the color of the film itself. Compared with the Control group, the conversion rate of oxygenated myoglobin to metmyoglobin was delayed in the other groups. This result may be related to the low oxygen permeability of CMC films and the antioxidant properties of CEO, Elhadef, K believes that adding antioxidants can delay the decline in a^*^ value (55).

#### 3.10.2 pH

The change of pH value of mutton is shown in Figure 5c. The pH value of mutton showed a trend of decreasing first and then increasing. On day 2, the mutton had the lowest pH value.

The decrease in pH values was because the blood circulation of the sheep was stopped after slaughtered. Anaerobic glycolysis made ADP rephosphorylated into ATP, during which lactic acid, H^+^ and energy were produced. The accumulation of lactic acid and H^+^ would reduce the pH value of the mutton. The subsequent rise in the pH value of mutton was caused by the breakdown of proteins resulting in accumulation of alkaline substances.

At 0–2 days, there were no significant differences in the pH values of mutton between the different groups. These results indicated that CMC-based films and CEO could not affect the anaerobic glycolysis of muscle glycogen. The pH value of the Control group dropped to the lowest point of 5.38 on day 2 and then rose to 6.23 (day 6), showing meat spoilage. At 0–12 days, there were no significant differences in pH values between the M0 and Control groups. The M1, M3, and M5 groups all delayed the rise in pH values of the mutton. With the increase in CEO concentration, the pH value increased less. This result may be due to inhibiting enzyme activity, microbial growth, and the production of nitrogen-containing compounds by adding CEO. With the increase in membrane antibacterial substance (CEO) content, the rise in pH value further slowed down, which had similar conclusions to other literature (1).

#### 3.10.3 TVB-N

TVB-N is an important index representing meat freshness, reflecting the degradation degree of protein and nitrogen-containing compounds in the meat itself. It is related to protease and spoilage bacteria (4). Figure 5d shows that on day 8, TVB-N in the Control group exceeded 15 mg/100 g, with a noticeable odor of corruption. There was no significant difference in TVB-N between the M0 and Control groups. The TVB-N accumulation rate in groups M1, M3, and M5 exhibited an inverse correlation with CEO concentration. Specifically, M1 and M3 exceeded the 15 mg/100 g threshold on the days 10 and 12, respectively, whereas M5 maintained sub-threshold levels throughout the 12day monitoring period. The addition of the CEO significantly slowed the rise in TVB-N. This result was caused by the excellent antibacterial performance of the CEO (Figure 3). This conclusion is similar to that previously reported (56).

In addition, the rise in pH affected the protease activity and proliferation of spoilage flora in meat. Some bacteria change from glycogen-dependent to protein-degrading types at high pH. The alkaline ammonia compounds produced by protein and putrefaction bacteria at high pH also increase the pH of meat, so TVB-N is positively correlated with pH values. This conclusion also explained that adding CEO could delay the rise of the pH values of mutton (Figure 5c).

#### 3.10.4 TBARS

MDA is the primary aldehyde produced by the oxidation of polyunsaturated fatty acids. It has a rancid odor, and its content in meat represents the degree of lipid oxidation (1). TBARS is one of the methods used to determine malondialdehyde content. As can be seen from Figure 5e, the TBARS value of the mutton in the Control group increased from 0.07 mg/kg on day 0 to 1.06 mg/kg on day 8, reaching the defining value of rotten meat. There was no significant difference in TBARS values between the M0 and Control groups. The TBARS values of M1, M3, and M5 groups on day 8 were 0.61, 0.58, and 0.38 mg/kg, respectively. The addition of CEO delayed the increase in TBARS values, indicating that CEO had a significant inhibitory effect on lipid oxidation. The concentration of CEO is directly proportional to its inhibitory effect on lipid oxidation.

Lipid oxidation is the result of many factors. In theory, the low oxygen permeability of M0 film could effectively isolate the contact between mutton and oxygen and reduce the oxidation rate of lipids. However, the result was not the opposite, which might be due to the excellent water solubility of CMC. After absorbing water, the structure of CMC films changed, and oxygen barrier performance worsened. A previous study found that increasing the water solubility of the film can reduce its oxygen barrier property and increase the lipid oxidation degree of shrimp meat, but adding antioxidants can significantly delay the lipid oxidation (57). CEO has excellent antioxidant capacity, which can inhibit and neutralize free radicals, compete with unsaturated fatty acids for oxygen, and achieve antioxidant effects. Therefore, adding CEO can protect unsaturated fatty acids from Lipid radicals and delay the rise of TBARS. Another way of lipid oxidation is Photooxidation. Unlike free radical oxidation of lipids, myoglobin or hemoglobin can directly generate hydroperoxides under light (3). With the increase in CEO concentration, the film can effectively block ultraviolet rays, prevent Photooxidation, and delay the lipid oxidation rate of mutton.

#### 3.10.5 TVC

The nutrients in mutton are conducive to the growth and reproduction of microorganisms and accelerate the rate of meat spoilage. As can be seen from Figure 5f, there was no significant difference in TVC values of mutton in the Control and M0 groups. The microbial limits for frozen meat were defined by the Chinese national standard GB 2707-2016, which specifies that the total aerobic bacterial count should not exceed 5.00 lg CFU/g. The TVC values on day 2 of these two groups of mutton was 5.42 lg CFU/g, exceeding the TVC threshold for spoilage meat. The time of M1, M3, and M5 groups exceeding the critical value was on days 4, 8, and 10, respectively. The concentration of CEO in the film was inversely proportional to the total number of viable bacteria in the mutton. This result could be attributed to the excellent antibacterial performance of the CEO. The CEO film could prevent mutton from being contaminated by external microorganisms. Its bacteriostatic mechanism was similar to the theory of bacteriostatic zone formation (Figure 3). The TVC values of the mutton coated with CEO films were significantly lower than that of the Control group, which was similar to the conclusion of a previous study (2).

#### 3.10.6 Odor analysis

**Figure 6:**
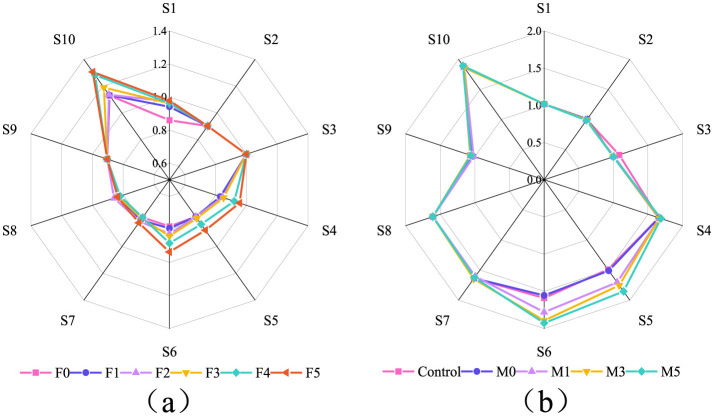
The odor responses from each sensor of the electronic nose: **(a)** for the films; **(b)** for the mutton wrapped with the films. CEO concentrations were 0, 1.5 g/L, 3.0 g/L, 4.5 g/L, 6.0 g/L, and 7.5 g/L for F0, F1, F2, F3, F4, and F5, respectively. The sample groups are the mutton covered with F0, F1, F3, and F5, which were shorted for M0, M1, M3, and M5, respectively. The mutton covered without film was shorted as control.

The distinct odor profile of CEO necessitated electronic nose analysis to characterize its impact on volatile aroma compounds in both composite films and mutton samples. Figure 6a displays the electronic nose response results of polymer composite films across different CEO concentrations. The incorporation of CEO significantly affected electronic nose responses to sulfur compounds (S4), nitrogenous compounds (S5), aldehyde/ketones compounds (S6), and short-chain alkanes (S10) in composite films, as signal intensity directly correlated with CEO concentration due to the bioactive constituents of CEO. These findings aligned with the sensory evaluation data from Supplementary Table S2. The F5 formulation exhibited unacceptable odor characteristics, whereas other composite films were within the acceptable thresholds.

Figure 6b displays the electronic nose response results of mutton samples packaged with composite films. Experimental data demonstrated that CMC-based films without CEO added had no significant effect on the volatile odor of mutton. Following CEO addition, sensor responses from S5 (nitrogenous compounds) and S6 (aldehyde/ketone compounds) displayed significant divergence and were proportional to the CEO loading level. Notably, the S10 (short-chain alkanes) sensor exhibited negligible response variations. This phenomenon likely originated from the masking effects of inherent short-chain alkane volatiles in mutton, which obscured analogous components released by the composite films (35). Comprehensive sensory evaluation results showed (Supplementary Table S2) that the volatile odor changes after composite films with different CEOs added were wrapped in mutton were primarily reflected in sulfide and nitrogen-containing compounds, with all detected odor intensities remaining within acceptable thresholds.

#### 3.10.7 Pearson correlation analysis

**Figure 7:**
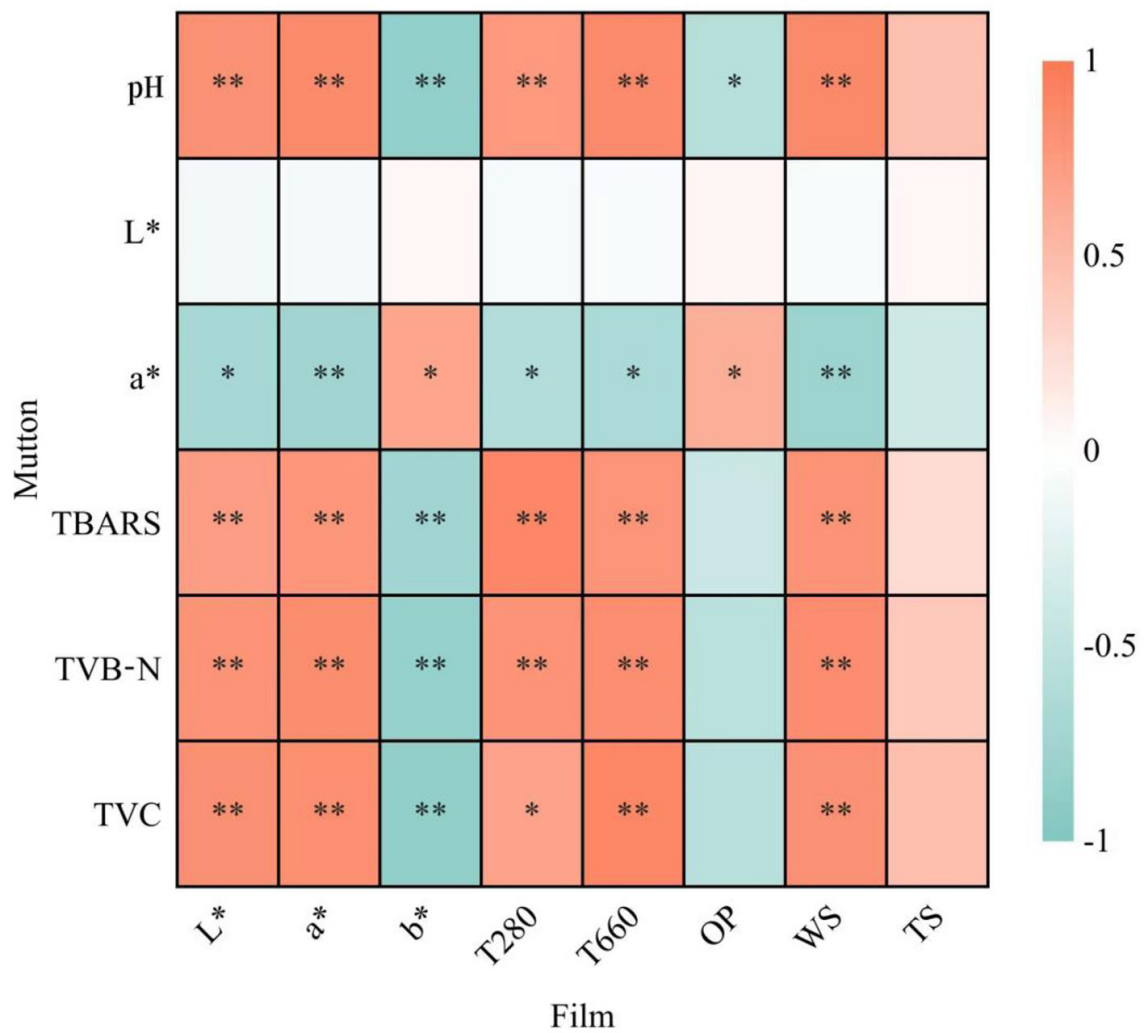
Correlation analysis between film parameters (L*, a*, b*, T280, T660, OP, WS, TS) and mutton quality parameters (pH value, L*, a*, TBARS, TVB-N, TVC). Asterisks indicate significance levels: *p ≤ 0.05, **p ≤ 0.01.

To better explain the influence of thin film on the shelf life of mutton, Pearson correlation analysis was used to evaluate the relationship between the mutton quality parameters (pH, L^*^, a^*^, TBARS, TVB-N, TVC) and thin film parameters (L^*^, a^*^, b^*^, T280, T660, OP, WS, TS) (Figure 7). The color parameters and visible light transmittance of the film were significantly correlated with pH values, TBARS, TVB-N, and TVC of mutton (|r| ≥ 0.8), which directly reflected that the concentration of CEO could affect the shelf life of mutton. In addition, the ultraviolet-blocking properties of the films were also significantly correlated with the pH values, TBARS, and TVB-N of the mutton (|r| ≥ 0.8). This result indicated that the ultraviolet irradiation increased the photooxidation degree of the mutton, thus affecting its shelf life. There was no correlation between the OP and TS of films and the shelf life of mutton (|r| ≤ 0.5). This result may be because the film absorbed water after contact with mutton, and the structure of the film changed, which also explained that the WS of films had a significant correlation with the shelf life of mutton (|r| ≥ 0.8). There was no correlation between the L^*^ value of the mutton and the film parameters (|r| ≤ 0.5), indicating that adding CEO could not affect the brightness of the mutton.

Collectively, chromatic aberration, WS, visible light transmittance, and UV-blocking efficiency of the composite films directly reflected CEO concentration gradients. Increased CEO loading induced marked deceleration in pH fluctuations, TBARS, TVB-N, and TVC trajectories during mutton storage, indicating CEO-mediated regulation effectively extended shelf life (|r| ≥ 0.5).

Advanced analysis revealed that practical applications of water-soluble edible films in meat preservation demand prioritized assessment of optical performance metrics and dissolution behavior, as these parameters directly govern the release kinetics of active compounds at the film-meat interface. OP and TS demonstrated constrained enhancement effects on meat preservation efficacy (|r| ≤ 0.5). This phenomenon stems from two mechanisms: elevated moisture content in meat products compromises the film's oxygen barrier functionality. The physical support from muscle tissue architecture reduces dependency on externally provided mechanical reinforcement. These findings underscore that in meat preservation systems, bioactive functionalities of packaging materials surpass conventional physical barriers (polyethylene/PE films) in preservation efficacy. Sun and YA also adopt a similar method to analyze the correlation between film and meat and reach a similar conclusion (36).

## 4 Conclusions

In this study, an edible composite film was prepared by adding CEO into the CMC film system, and it could delay the decline of meat quality during storage. The addition of the CEO made the film show good antibacterial properties, high visible light transmittance, and excellent ultraviolet blocking properties. In contrast, MA and WS decreased, and the thickness of the film increased. FT-IR and XRD results showed that adding CEO did not produce a new chemical and crystal structure but reduced the density of the film. All film groups achieved complete soil biodegradation within 12 days, enabling sustainable utilization of biomass resources. Compared with mutton samples packaged without film lamination or control films, CEO-incorporated films significantly retarded spoilage progression and enhanced color attributes in refrigerated mutton (4°C) during a 12-day storage period. The electronic nose and sensory evaluation concluded that the mutton smell changed after coating, but the altered odor was all within the acceptable range. Finally, Pearson correlation analysis showed that the color difference, ultraviolet blocking property, and WS of the films had a significant correlation with the mutton spoilage index. The OP and mechanical properties could not affect the shelf life of the mutton.

The results demonstrated that CEO composite films exhibited resource-efficient, eco-friendly, and biodegradable characteristics aligning with environmental sustainability requirements in food packaging while also demonstrating the feasibility of extending mutton shelf life through CMC composite films containing CEO. However, this study had limitations, including challenges in the large-scale industrial production of composite films and the need to balance the cost-effectiveness of CEO incorporation in films.

## Data Availability

The original contributions presented in the study are included in the article/Supplementary material, further inquiries can be directed to the corresponding authors.
