# Supplementary material for: Preparation and characteristics of carboxymethyl cellulose-based films embedding cinnamon essential oil and their application on mutton preservation
Source: Front Nutr. 2025 Apr 30;12:1559833. doi: 10.3389/fnut.2025.1559833 (PMC12075238; doi:10.3389/fnut.2025.1559833)
Supplement: Supplementary file 1 [file Data_Sheet_1.pdf]

Supplementary Material

1 Material and methods

1.1 Material

The CEO was supplied by FLOWERS & HERBS Co. Ltd., Beijing, China, and produced by steam distillation of fresh cinnamon tree branches, leaves, and bark. The main components of CEO are Cinnamaldehyde (80.12%), Eugenol (6.25%), Cinnamyl acetate (1.15%), β-Caryophyllene (1.10%), and Benzaldehyde (0.67%).

1.2 Measurement of minimum inhibitory concentration (MIC) and minimum bactericidal concentration (MBC).

The MIC and MBC were measured following the method described in a previous study with some modifications(Wen et al., 2016). Microorganisms were inoculated in nutrient broth at 37 ℃ for 24h and diluted to 0.5 McFarland standards. 0.5 mL microbial culture medium (0.5McFarland standard) was added into 10 mL emulsion with different CEO concentrations (prepared as the method described in 2.2) and cultured at 37°C for 24 h to observe turbidity. Colony growth is positive (+). Aseptic growth is negative (-), and the lowest concentration of CEO lotion is MIC. According to the MIC results, 100 μL of the mixed bacterial solution was cultured in the solid medium at 37°C for 24h, and MBC was obtained if no colony was produced.

1.3 Sensory evaluation

Ten trained panelists were recruited to conduct sensory evaluations on both composite films and film-coated lamb samples (Khazani et al., 2024). All panelists were proficient in sensory evaluation techniques. A 5-point hedonic scale was used (1=unacceptable, 3=acceptable, 5=extremely preferred). Evaluations were conducted in a standardized sensory laboratory, with purified water rinsing and a 10-minute rest period between sample evaluations to eliminate sensory carryover effects.

2 The results and discussion

2.1 MIC and MBC

Given the contrary conclusion on the bacteriostatic strength of Escherichia coli and Staphylococcus aureu with the addition of CEO in the CMC-based films, this study continued to determine the MIC and MBC of the film-forming solution. Here is a list of the opposite conclusions: Ruimin Ran mixed the CEO and soy protein isolate to form a film, and the antibacterial effect of Escherichia coli was stronger than that of Staphylococcus aureus, which was interpreted as that the CEO may contain certain compounds with specificity that can recognize the target of the outer membrane of Escherichia coli. Thus, the substances penetrated bacterial cells(Ran et al., 2023).

Table S1 shows that when the concentration of CEO in the emulsion was 1.5 g/L, the emulsion had an antibacterial effect, and the turbidity of the test tube could be observed to disappear. *Escherichia coli* and *Staphylococcus aureus* growth were not observed at CEO concentrations of 3.5

g/L and 2.0 g/L. According to MBC results, CEO was more effective against *Staphylococcus aureus* than *Escherichia coli*. The experimental results verified the change in antibacterial zone diameter. Similar conclusions are obtained in previous literature(Yang et al., 2021).

3 Supplementary Table

Table S1 MIC and MBC of films without CEO or with different CEO concentration.

CEO concentration in emulsion (g/L)	*E. coli*	*S. aureus*
0	+[a]	+
1.0	+	+
1.5	-[b]	-
2.0	-	-
2.5	-	-
3.0	-	-
3.5	-	-
MIC	1.5	1.5
MBC	3.5	2.0

+[a], colony growth is positive

-[b], aseptic growth is negative

Table S2 Sensory evaluation of films with varying CEO concentrations and the mutton wrapped with corresponding CEO-incorporated films

Sample		Sensory parameters (hedonic score 1-5)			
		Color	Smell	Softness	General Acceptance
Films	F0	4.76 ± 0.24^{e}	4.29 ± 0.24^{c}	2.87 ± 0.32^{b}	4.35 ± 0.34^{c}
	F1	4.33 ± 0.21^{d}	4.63 ± 0.29^{d}	3.68 ± 0.52^{c}	3.86 ± 0.26^{bc}
	F2	3.71 ± 0.30^{c}	3.35 ± 0.21^{b}	3.53 ± 0.42^{bc}	3.64 ± 0.45^{ab}
	F3	3.25 ± 0.15^{b}	3.37 ± 0.25^{b}	3.37 ± 0.31^{bc}	3.55 ± 0.32^{ab}
	F4	3.16 ± 0.13^{b}	3.17 ± 0.28^{b}	3.12 ± 0.38^{bc}	3.30 ± 0.28^{ab}
	F5	2.25 ± 0.34^{a}	1.94 ± 0.65^{a}	2.04 ± 0.28^{a}	3.06 ± 0.23^{a}
Mutton	Control	3.15 ± 0.34^{b}	4.39 ± 0.42^{c}	3.52 ± 0.38^{d}	3.33 ± 0.25^{b}
	M0	3.26 ± 0.23^{b}	4.42 ± 0.34^{c}	3.13 ± 0.32^{bc}	3.31 ± 0.18^{b}
	M1	3.03 ± 0.16^{b}	4.31 ± 0.35^{c}	2.78 ± 0.22^{bc}	3.12 ± 0.26^{b}
	M3	2.86 ± 0.20^{b}	3.33 ± 0.25^{b}	2.57 ± 0.51^{ab}	3.05 ± 0.17^{b}
	M5	2.16 ± 0.32^{a}	2.01 ± 0.57^{a}	2.03 ± 0.32^{a}	2.38 ± 0.34^{a}

Data were presented as mean ± standard error. CEO concentrations were 0, 1.5 g/L, 3.0 g/L, 4.5 g/L, 6.0 g/L, and 7.5 g/L for F0, F1, F2, F3, F4, and F5, respectively. The sample groups are the mutton covered with F0, F1, F3, and F5, which were shorted for M0, M1, M3, and M5, respectively. The mutton covered without film was shorted as Control. a-e Different superscripts indicate significant differences in the same column ($P \leq 0.05$).

4 Reference

Khazani, B., Almasi, H., Mohtarami, F., and Amjadi, S. (2024). Incorporation of Artemisia essential oil loaded chitosomes in salep based film for use in toast bread packaging: New generation of active films. *Food Packaging and Shelf Life* 43**,** 101305. doi: 10.1016/j.fpsl.2024.101305.

Ran, R.M., Zheng, T.T., Tang, P.P., Xiong, Y.M., Yang, C.K., Gu, M., et al. (2023). Antioxidant and antimicrobial collagen films incorporating Pickering emulsions of cinnamon essential oil for pork preservation. *Food Chemistry* 420. doi: 10.1016/j.foodchem.2023.136108.

Wen, P., Zhu, D.H., Wu, H., Zong, M.H., Jing, Y.R., and Han, S.Y. (2016). Encapsulation of cinnamon essential oil in electrospun nanofibrous film for active food packaging. *Food Control* 59**,** 366-376. doi: 10.1016/j.foodcont.2015.06.005.

Yang, K., Liu, A.P., Hu, A.X., Li, J.X., Zen, Z., Liu, Y.T., et al. (2021). Preparation and characterization of cinnamon essential oil nanocapsules and comparison of volatile components and antibacterial ability of cinnamon essential oil before and after encapsulation. *Food Control* 123. doi: 10.1016/j.foodcont.2020.107783.
